# Factors affecting the support for physical activity in children and adolescents with type 1 diabetes mellitus: a national survey of health care professionals’ perceptions

**DOI:** 10.1186/s12887-023-03940-3

**Published:** 2023-03-22

**Authors:** Emma J. Cockcroft, Eva L. Wooding, Parth Narendran, Renuka P. Dias, Alan R. Barker, Christopher Moudiotis, Ross Clarke, Robert C. Andrews

**Affiliations:** 1 University of Exeter Medical School, Exeter, EX1 2JP UK; 2 Department of Paediatrics, Royal Devon University Healthcare NHS Foundation Trust, Exeter, EX2 5DW UK; 3 Department of Diabetes, University Hospitals Birmingham NHS Foundation Trust, Birmingham, UK; 4 Institute of Immunology and Immunotherapy, University of Birmingham, Birmingham, UK; 5 Department of Endocrinology and Diabetes, Birmingham Children’s Hospital, Birmingham Women’s, and Children’s NHS Foundation Trust, Birmingham, UK; 6 Children’s Health and Exercise Research Centre, Sport and Health Sciences, University of Exeter, Exeter, EX1 2JP UK; 7 Department of Diabetes, Taunton and Somerset NHS Foundation Trust, Taunton, UK

**Keywords:** Intervention development, Behaviour change, Young people, Exercise

## Abstract

**Background:**

Many children and adolescents with Type 1 Diabetes Mellitus (T1DM) don’t meet the recommended levels of physical activity. Healthcare professionals (HCPs) have a key role in supporting and encouraging children and adolescents with T1DM to be physically active. This study aims to understand the perspectives of HCPs in relation to supporting physical activity and implementing guidelines relating to physical activity.

**Methods:**

An online mixed methods survey was circulated to HCPs in pediatric diabetes units in England and Wales. Participants were asked about how they support physical activity in their clinic and their perceptions of barriers/enablers of providing physical activity support to children and adolescents with T1DM. Quantitative data were analysed descriptively. An deductive thematic approach was applied to the free text responses using the Capability Opportunity Motivation model of Behaviour (COM-B) as a framework.

**Results:**

Responses were received from 114 individuals at 77 different pediatric diabetes units (45% of pediatric diabetes units in England and Wales). HCPs surveyed felt that the promotion of physical activity is important (90%) and advised patients to increase levels of physical activity (88%). 19% of the respondents felt they did not have sufficient knowledge to provide support. HCPs reported limited knowledge and confidence, time and resources as barriers to providing support. They also felt the current guidance was too complicated with few practical solutions.

**Conclusion:**

Pediatric HCPs need training and support to be able to encourage and support children and adolescents with T1D to be a physical activity. In addition, resources that provide simple and practical advice to manage glucose around exercise are needed.

**Supplementary Information:**

The online version contains supplementary material available at 10.1186/s12887-023-03940-3.

## Introduction

Type 1 Diabetes Mellitus (T1DM) is one of the most common chronic conditions in children and adolescents [1]. The management of T1DM requires the administration of exogenous insulin, the dose of which is adjusted according to blood glucose values and dietary intake [2]. Regular physical activity is also important in the management of T1DM and is associated with improvements in metabolic control, body composition, quality of life, and mental wellbeing, as well as protecting against future development of cardiovascular disease and premature mortality [3–5]. As a consequence of these benefits, physical activity is explicitly mentioned in National Institute for Health and Care Excellence (NICE) guidance for the management of children and young people with T1DM. Despite the benefits and clinical recommendations, studies suggest that up to 70% of children and adolescents with T1DM are not meeting the recommended 60 min of moderate to vigorous physical activity per day [6] and are less physically active compared to peers without T1DM [7]. Children and adolescents with T1DM are likely to find physical activity recommendations harder to achieve than those without T1DM due to the unique difficulties associated with the management of their condition, particularly fear of hypoglycemia around exercise, lack of knowledge on how to manage blood glucose during exercise, and the need to check blood glucose levels during exercise [8].

Behaviour is not just influenced by the individual and changing behaviour requires change in multiple domains. Healthcare professionals (HCPs), such as senior doctors and specialist nurses working within paediatric diabetes clinics have a central role in ensuring recommendations around physical activity are supported and encouraged and help with behavioural change. The need for this support is highlighted by the International Society for Paediatric and Adolescent Diabetes (ISPAD), who have issued consensus guidelines for exercise in children and adolescents with T1DM [9]. These recommendations include clinical guidelines around supporting physical activity in clinics. It is currently not known if paediatric clinics in England and Wales are aware of these recommendations. Nor do we know what factors affect HCPs support for physical activity in their patients.

Theoretical models can provide a framework for understanding influences on behavior. The Capability, Opportunity, and Motivation model of Behavior (COM-B) presents human behavior as resulting from interactions between ‘capability’ (physical and psychological), ‘opportunity’ (physical and social), and ‘motivation’ (autonomic and reflexive) [10]. Detailing factors influencing behaviours falling under the three COM-B components can be used to understand potentially modifiable factors to target in an intervention.

Previous research has explored factors affecting children and adolescents with T1DM from the perceptions of HCPs [8], with a focus on barriers for children and adolescents and not necessarily factors which may influence the support HCPs can provide. The purpose of this study is to build on this previous work to understand the factors which influence HCPs’ ability to support physical activity and implement ISPAD guidance.

## Methods

A 14-question cross-sectional survey hosted by Jisc Online survey software (https://www.onlinesurveys.ac.uk) was developed using a mixture of closed and open questions. The survey aimed to ascertain perceived barriers to HCP, support of meeting, recommended physical activity targets, and patients’ engagement in this. Demographic data were also collected on the size and location of the pediatric diabetes clinic and the professional role of the people completing the survey. Participants were asked to rate statements on a five-point Likert scale from ‘strongly agree’ to ‘strongly disagree’ on a range of themes designed to ascertain their views about their role as professionals in supporting young people with diabetes to be more active, the barriers to supporting them, their awareness of guidance informing best practice, and their views on what would facilitate increased physical activity in their patient population. A copy of the survey is included in Supplementary materials.

The content of the survey was based on the recommendations from ISPAD on physical activity for young people with T1DM and building on previous unpublished work in adults with T1DM by members of the study team. The survey was piloted by five independent HCPs prior to circulation, who suggested changes in structure and wording.

### Sample

Survey data were collected from September 2019 to June 2020. All 173 pediatric diabetes units in England and Wales were invited to participate via email from regional representatives of the National Children and Young People Diabetes Network. This was supplemented by social media posts and snowball sampling amongst colleagues. The participants were a convenience sample of consented participants from those units. All HCPs working in paediatric diabetes units were eligible to participate. Because of the nature of participant recruitment, it was not possible to determine the total number of HCPs approached to take part in this study.

### Data analysis

Survey responses were downloaded from survey software and anonymised. Quantitative responses were exported into SPSS (version 22.0, Chicago, USA) and analysed descriptively. Where relevant, categorical data were presented as frequencies. Survey responses were checked by the first author to ensure they met the inclusion criteria of being adequately filled out (< 10 missing items) by HCPs treating children and adolescents with T1DM. No responses were excluded.

Survey responses for the open-ended qualitative questions were analysed using a deductive thematic analysis using the COM-B behavior change framework [10]. The thematic approach suits questions relating to experiences, views or perceptions and is a commonily used methods for identifying and reporting within qualitative data [11].

Free text responses were imported into NVivo (version 12) for analysis. Two researchers (EC, EW) initially familiarized themselves with the free text responses before independently coding responses to the COM-B domain that they were judged to represent. Similar responses in the same COM-B domain were grouped and given a label which summarised the factors influencing behaviour. Each stage of analysis was conducted independently by two researchers (EC & EW) with uncertainties resolved through discussion.. Key factorss were described as either barriers; problems, issues, challenges, and/or difficulties with physical activity participation or support; or enablers; programmes, interventions, or factors that may improve physical activity participation or support.

Findings from quantitative and qualitative open ended responses were synthesized to evaluate targets for change to improve how HCPs can support physical activity. The sysnthesis was guided by the COM-B and Theoretical Domains Framework (TDF) to facilitate organization of influencing factors and potential intervention functions.

## Results

### Sample characteristics

One hundred fourteen responses were received from HCPs working at 77 different paediatric diabetes units (45% of paediatric diabetes units in England and Wales [34% (*n* = 39) specialist nurses, 27% (*n* = 31) dieticians, 31% (*n* = 35) senior doctors, 7% (*n* = 8) psychologists, 1% (*n* = 1) associate specialists].

**Fig. 1:**
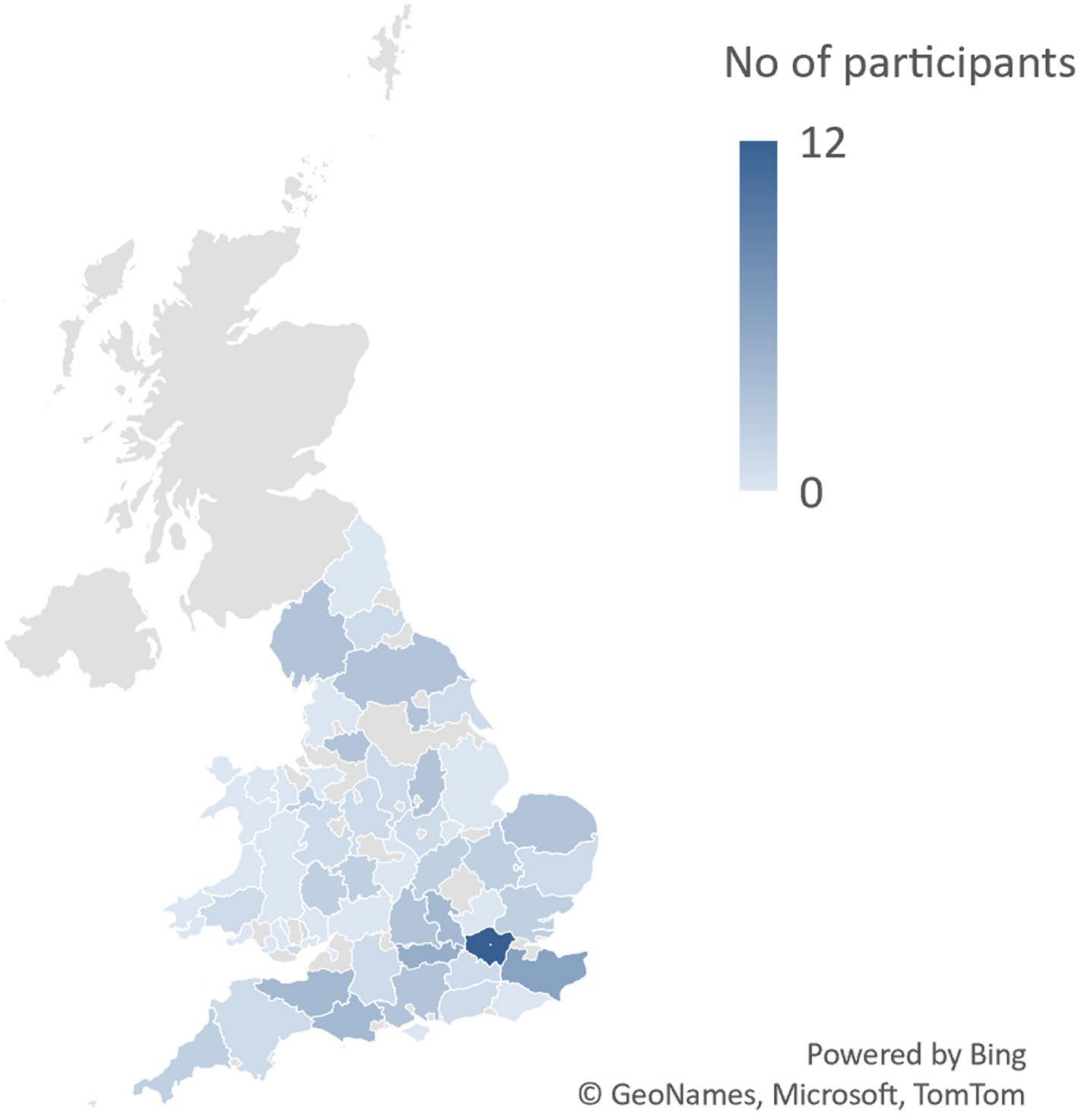
Distribution of respondents by county

44% (*n* = 50) of participants worked in clinics with over 200 patients on their caseload. Another 22% (*n* = 25) of the respondents worked in clinics with 150–199 patients and 24% (*n* = 27) in clinics with 100–149 patients. Only 11% of professionals worked in smaller clinics with 51–99 patients. Clinics were spread across different regions with the highest number of responses were from HCPs working in the Southeast of England (26%, *n* = 30) and the Northeast and Yorkshire (21%, *n* = 25).The distributions of respondents split by county are shown in Fig. 1.

79% (*n* = 90) of participants were aware of ISPAD guidance around supporting physical activity, of which 67% (*n* = 76) found the guidance useful. 61% (*n* = 70) of respondents had received a type of training around supporting physical activity. This varied from formal academic qualifications (e.g., MSc degree), conferences (such as EXTOD and PEAK), and informal advice and support from colleagues.

### HCP’s attitude towards supporting physical activity

HCPs attitudes towards supporting physical activity are shown in Table 1, responses split by role are shown in Supplementary file 1. Most respondents felt that supporting (94%) and promoting (89%) physical activity in their patients was part of their clinical role. 12 respondents (11%) were neutral or disagreed that promoting physical activity was important. 96% of said that blood glucose management plans during exercise were part of an ongoing education programme for patients (96%) with advice generally given even when unprompted by patients. Most respondents (80%) felt they had sufficient knowledge to provide advice around physical to their patients. However, 22 respondents (19%) did not feel they have sufficient knowledge to advise children and adolescents with T1DM about physical activity.

**Table 1:** HCPs views and attitudes towards supporting physical activity in clinics. *N* = 114

item	Strongly agree, n (%)	Agree, n (%)	Neither agree nor disagree, n (%)	Disagree, n (%)	Strongly disagree, n (%)
Helping children and adolescents with type 1 diabetes to be physically active is part of my clinical role	74 (65%)	33 (29%)	5 (4%)	2 (2%)	0 (0%)
Promoting physical activity in children and adolescents with type 1 diabetes is seen as important in the clinic that you work in	65 (57%)	37 (32%)	10 (9%)	2 (2%)	0 (0%)
I don't advise children and adolescents with type 1 diabetes about physical activity unless specifically asked by the patient	1 (1%)	6 (5%)	5 (4%)	53 (46%)	49 (43%)
I don't advise children and adolescents with type 1 diabetes about physical activity unless the patient reports difficulties with physical activity	0 (0%)	6 (5%)	4 (4%)	57 (50%)	47 (41%)
I have sufficient knowledge to advise children and adolescents with type 1 diabetes about physical activity	30 (26%)	62 (54%)	15 (13%)	5 (4%)	2 (2%)
I try to encourage children and adolescents with type 1 diabetes to increase their physical activity levels	42 (37%)	58 (51%)	11 (10%)	3 (3%)	0 (0%)
When physical activity is discussed, advice is given to children and adolescents with type 1 diabetes on managing blood glucose	81 (71%)	30 (26%)	3 (3%)	0 (0%)	0 (0%)
Blood glucose management during exercise is included as part of the ongoing education programme for children and adolescents with type 1 diabetes	76 (67%)	33 (29%)	4 (4%)	1 (1%)	0 (0%)

### Factors affecting physical activity support in clinic

Responses to questions relating to factors affecting physical activity support in clinic are shown in Table 2. Most respondents (82%) felt confident in giving advice to their patients, but a large proportion of respondents (44%) felt there was a lack of educational opportunities for HCPs relating to supporting physical activity in their patients. Only 52% thinking that the current provision of educational materials for patients was appropriate.

**Table 2:** HCPs views on factors affecting physical activity support in clinic

item	Strongly agree, n (%)	Agree, n (%)	Neither agree nor disagree, n (%)	Disagree, n (%)	Strongly disagree, n (%)
I do not have enough time to discuss physical activity with children and adolescents with type 1 diabetes	0 (0%)	16 (14%)	25 (22%)	54 (47%)	19 (17%)
Educational materials about physical activity are inappropriate for children and adolescents with type 1 diabetes	4 (4%)	18 (16%)	33 (29%)	44 (39%)	15 (13%)
There is a lack of educational opportunities for health professionals regarding physical activity in children and adolescents with type 1 diabetes	8 (7%)	42 (37%)	24 (21%)	37 (32%)	3 (3%)
Children and adolescents with type 1 diabetes are unlikely to be motivated to follow advice to be more active	1 (1%)	17 (15%)	34 (30%)	47 (41%)	15 (13%)
I feel confident giving children and adolescents with type 1 diabetes advice on managing blood glucose with physical activity	36 (32%)	57 (50%)	12 (11%)	15 (13%)	3 (3%)

### Barriers and enablers of supporting physical activity

**Fig. 2:**
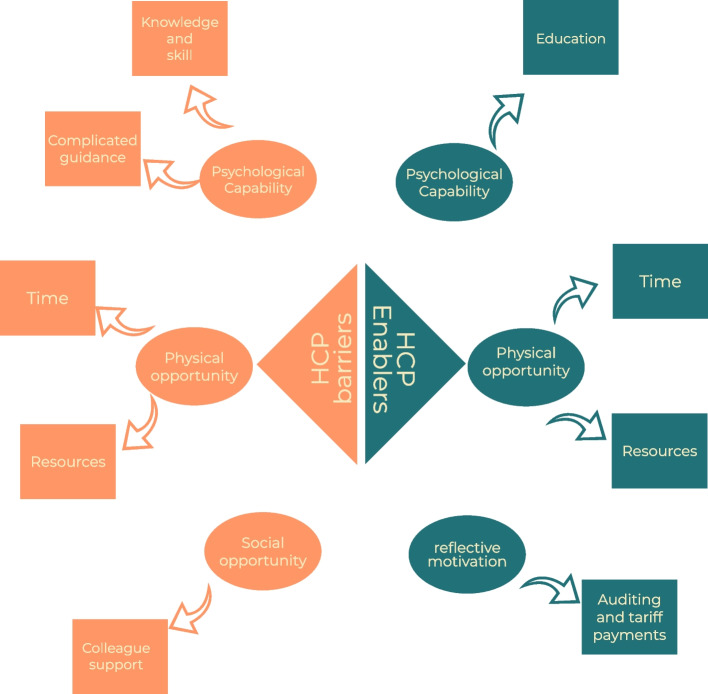
Summary of HCP level barriers and enablers to supporting physical activity

From the free text survey responses, we identified a a number of factors within each COM-B domain.thefrequency, whether they are barriers or enablers, along with supporting quotes are presented in Table 3 and summarised in Fig. 2.

**Table 3:** Summary of COM-B themes classified and Barriers and Enablers for health care professionals

COM-B component	Factor	Barrier/enabler	Frequency	Sample Quote
Psychological Capability	Knowledge and skill	Barrier	15	*“no support/programmes/resources to support children with physical activity”* *“no formal qualification. unsure of ability to suggest insulin dose changes”* *“A lack of training around how to manage blood glucose levels with activity—this is not standard training given to psychologists”*
	Complicated guidance	Barrier	4	*“No explicit guidance is given around the practicalities of what exercise to promote and when and how to review this in a professional capacity”*
	Education	Enabler	50	*“Better training and local diabetic networks to work on agreed consensus”* *“National guidance—e.g. flow chart or info sheet to provide to families Making it auditable as to whether it is discussed.”* *“opportunity to network attend education specific to diabetes, type 1 and CYP”*
Physical opportunity	Resources	Barrier	10	*“Making it easier to understand no support/ programmes/ resources to support children with physical activity”* *“I feel able to offer appropriate advice if they desire it, but if I am seeking to persuade them to increase exercise (i.e. change their behaviour) this requires intensive coaching-style support which we do not have resource to offer.”*
	Resources	Enabler	38	*“Places to signpost children and adolescents to—lots of sites available but not very child focused”* *“Simplified instructions Electronic resources to direct patients to”*
	Time	Barrier	37	*“Sometimes there isn't adequate time in clinic.”* *“Having specific time to spend a long discussion in the clinic setting.”*
	Time	Enabler	15	*“Time to discuss these issues,”* *“To acknowledge that this topic is required to be discussed as one of the important factors and one of the basics of the diabetes care. Have available time to support 'fine tuning' of advice.”*
Social opportunity	Colleague support	Barrier	6	*“lack of support from colleagues and no formal qualification.”* *“consistent team approach. support from colleagues and no formal qualification. unsure of ability to suggest insulin dose changes”*
Reflective motivation	Auditing and Tarif payments	Enabler	5	“Make this a mandatory part of diabetes care e.g. inclusion of PA measurement and support in the National Paediatric Diabetes Audit.”

### HCP barriers/enablers to supporting physical activity

#### Psychological capability

Two barriers to HCP support for physical activity were identified: (1) *knowledge and skills* and (2) *complicated guidance.* Knowledge and skills related to the HCPs ability and confidence in providing support for young people. Several respondents suggested that the lack of formal qualifications and training could inhibit their ability to provide support: “*no formal qualification. unsure of the ability to suggest insulin dose changes” (Dietitian).* This barrier was coupled with current *complicated guidelines: “No explicit guidance is given around the practicalities of what exercise to promote and when and how to review this in a professional capacity”. (Psychologist).*

The provision of *knowledge and education* was frequently suggested as an enabler to the provision of support for physical activity. One HCP stated *“Better training and local diabetic networks to work with an agreed consensus” (senior doctor)* as an approach to improve support.

#### Physical opportunity

*Time* and *resources* were identified as both barriers and enablers to support physical activity. In terms of resources, HCPs would value somewhere to direct patients to simplified instructions and child-focused information. The lack of appropriate resources at present was noted as a barrier, for example, one participant noted: *“Making it easier to understand no support/ programmes/ resources to support children with physical activity” (specialist nurse).* The second theme relating to physical opportunity was *time.* It was noted that *“there isn’t adequate time in clinic” (Dietitian)* and that it would be beneficial to have “time to discuss these issues” (senior doctor) and to “have available time to support the fine tuning of the advice” (dietitian).

#### Social opportunity

C*olleague support*, was noted as a barrier to supporting physical activity. There was a “lack of support from colleagues” (dietitian) and wasn’t a “consistent team approach” (dietitian).

#### Reflective motivation

Auditing and tariff payments were suggested as an enabler for supporting physical activity. *“Make this a mandatory part of diabetes care, e.g. inclusion of physical activity measurement and support in the National Paediatric Diabetes Audit” (dietitian).*

### Synthese of findings – interventions suggestions

From the synthesis of findings we identified 5 intervention functions which were relevant for enabling HCPs to support physical activity in adolescents with T1DM. The synthesis and links between the COM-B model, TDF and intervention functions are shown in Table 4.

**Table 4:** Synthesis of findings. Links between COM-B model, TDF domains and suggestions of intervention functions to enable HCPs to support physical activity in adolescents with T1DM

barriers and enablers for supporting adolescents with T1DM to be physically active		TDF domains linking to COM-B components	Possibly intervention functions
CAPABILITY	*Psychological Capability:*		
	Limited knowledge on what to advise in terms of adjustments to insulin and carbohydrate intake, as well as how different forms of exercise may impact blood glucose levels	Knowledge and skills	*Education, Training* – increasing HCP knowledge and understanding of physical activity as well as skills training to help them motivate and encourage behaviour change in adolescents they care for
OPPORTUNITY	*Physical opportunity:*		
	Time	Environmental context and resources	*Environmental restructuring* – Ensure HCPs have time to have discussions around physical activity or prioritise these conversations
	Resources – have the necessary materials to be able to signpost to	Environmental context and resources	*Enablement* – Provide resources to allow HCPs to signpost to advice. Ensure guidance is easy to follow and accessible
	Current guidelines that HCPs are meant to follow are Complex and so hard to implement in practice		
	*Social opportunity:*		
	Colleague support – having support from others to do it and people around them doing it	Social influences	*Modelling, Enablement* – Making conversations about physical activity a normal part of consultations. Having physical activity champions within clinics
MOTIVATION	**Reflective motivation:**		
	Important part of clinical role – Have a sense that they should be doing it	Memory, attention and decision Processes	*Training, environmental restructuring –* Enuring that supporting physical activity remains an important part of clinical role through training and providing prompts to have conversations with patients
	Auditing and tariff payments	Reinforcement	*Incentivisation, Environmental restructuring –* Making support for physical activity part of clinical care and auditable, or incentivising promotion of physical activity
	**Automatic motivation:**		
	Confidence	Beliefs about capabilities	*Education, Modelling – education to boost confidence in providing evidence based advice to patients*

## Discussion

The results from this study provide valuable insight into current practice and how children and adolescents with T1DM can be better supported to achieve physical activity guidelines, as well as how HCPs can be assisted to enable this change in behavior. This study used the COM-B theoretical framework of behavior to understand the potential barriers and enablers to physical activity support from the perspective of HCPs working in paediatric diabetes units. In the present study, we found that the majority of HCPs valued the importance of promoting and supporting their patients to be physically active, despite highlighting a number of factors that influence their ability to support patients with this.

To our knowledge, this is the first study which has used the COM-B theoretical framework to understand both barriers and enablers to supporting physical activity from the perspective of HCPs, helping to situate the findings within a frameowork of behvioural theory to develop an intervention to address the identified barriers. Previous studies have investigated the perspective of patients [12–15] their families [16–18], and HCPs perspectives of children and adolescents’ barriers [19], but not specifically their own barriers to support. This topic has recently been reviewed by Dash and colleagues (2020) [8]. Given the role of HCPs in supporting children and adolescents with T1DM [20], a theoretical understanding of factors which may inhibit and enable better support is vital to try and address the current low levels of physical activity within this population. Our results highlight a number of factors across the COM-B domains which can be targeted in future interventions which aim to improve physical activity in children and adolescents with T1DM.

The majority of respondents reported that supporting and promoting physical activity was part of their clinical role (95% and 80% respectively), however approximately 1 in 5 respondents felt they did’t have sufficient knowledge. These findings are in line with previous work from Ilkowitz and colleagues [21] in the USA. In their sample, 85.5% of providers believed that counselling regarding exercise for pediatric patients was a priority. Interestingly, this study also highlighted the limited knowledge of providers around physical activity guidelines with 79.3% of respondents not able to correctly identify American Diabetes Association guidelines correctly. This goes beyond our data which is limited to self-reporting of own knowledge and confidence levels instead of more formal ‘testing’. Data from the present study also suggest that 80% of HCPs are aware of ISPAD guidelines, with 67% of those aware finding them helpful. Given the importance of physical activity, it is important to raise awareness of the use of this available guidance and work with staff to ensure it is in an accessible and helpful format to be implemented in clinics.

Despite respondents suggesting they are able to provide support for children and adolescents with T1DM as part of their clinical role, the findings highlight a number of barriers relating to this support. Acknowledging these barriers is important as support received from professionals has been shown to facilitate participation in physical activity [22]. The main aspect of psychological capability was the acknowledgement that there were limited support and training opportunities for HCPs, with no formal qualifications and no explicit guidelines around the practicalities of promoting physical activity. Only 26% of respondents reported to strongly agree that they had sufficient knowledge relating to this and HCP education was frequently suggested as an enabler to supporting children and adolescents with T1DM. These findings suggest the importance of developing and providing formal training to staff and have clear national guidelines to help enable support for children and adolescents with T1DM. The suggestion of the importance of education has been previously discussed by others [19], but with focus on patient and parent education rather than specifically on HCPs. Given the important role of HCPs in supporting patient care, we would argue that both are essential components of supporting physical activity.

Quantitative responses to this survey suggest that most HCPs had adequate time, however responses to free text questions indicate that HCPs perceive time to be a barrier to providing specific support for children and adolescents with physical activity. The idea of time was often related to complex cases and fine tuning advice, rather than giving simple generic information. The idea of lack of time has previously been described elsewhere in a qualitative synthesis of factors that affect participation in physical activity among children and adolescents with T1DM [8], where it was stated that current deliverers of education may have a lack of time to cover all areas. It was suggested by a number of participants in our study that including an assessment of physical activity in tariff payments or in the National Pediatric Diabetes Audit could help to ensure physical activity is supported and monitored in clinics. We suggest this could be in an easy-to–to-implement format of the physical activity questionnaire or more formal objective measurements of physical activity with accelerometers at annual reviews.

It is important to acknowledge that HCPs promotion physical activity and providing advice to children and adolescents is part of a complex system of behaviours. In isolation improving the support for HCPs is only part of the solution and may not alone increase physical activity levels of children and adolescents with T1DM. As previously reported by others [8] factors such as motivation of children and adolescents to be physically active, fear of hypoglycemia and social support are also important factors and need to be considered alongside any HCP focused intervention. An additional consideration is the complexity of support, with different recommendations and lack of evidence based consensus on adjustments to insulin (CSII and MDI), carbohydrate intake and the effects of different exercise types and intensities of blood glucose specifically for children and adolescents [23].

The findings of the current study should be considered in light of several limitations. Firstly, the convenience sample of this survey means that HCPs were self-selected and may not be representative. HCPs with more experience of supporting physical activity or who value the importance of physical activity may have been more likely to complete this survey, reducing the generalizability of the results. However, a range of opinions seem to be captured and our sample has highlighted a number of barriers which add to the existing body of research in this area. Secondly, the use of survey methodology meant we could not explore the suggested barriers in depth with the researcher unable to follow up ideas and clarify issues. However, this methodology has allowed us to gain insight into individuals' perspectives and experiences [24] in a relatively large sample of HCPs across England and Wales, reaching 44% of paediatric diabetes units.

## Conclusions and recommendations

Our findings suggest that HCPs feel that they need more specific training and support to enable them to give advice to children and adolescents. Findings from this study suggest that developing a HCP focused educational rources to help increase knowledge and understanding around physical activity and T1DM would be a useful approach to help HCPs to provide improved support for children and adolescents with T1DM. Future research should develop a more indeph understanding of the educational needs of HCPs as well as how best to provide this support in terms of content, format and implementation considerations such as time to undertake any training. Additionally findings suggest the potential impact of developing patient friendly resources to help in their support as well as incentivisation, for example, by including physical activity support as part of the paeadiatric best practive tarif.

## Supplementary Information

**Additional file 1:** **Table S1.** HCPs views and attitudes towards supporting physical activity in clinics. *N*=114. Split my role. **Table S2.** HCPs views on Factors affecting physical activity support in clinic, split by role.

## Data Availability

The datasets generated and/or analysed during the current study are not publicly available but are available from the corresponding author on reasonable request.
