# Supplementary material for: Factors affecting the support for physical activity in children and adolescents with type 1 diabetes mellitus: a national survey of health care professionals’ perceptions
Source: BMC Pediatr. 2023 Mar 22;23:131. doi: 10.1186/s12887-023-03940-3 (PMC10031957; doi:10.1186/s12887-023-03940-3)
Supplement: Supplementary file 1 — Additional file 1: Table S1. HCPs views and attitudes towards supporting physical activity in clinics. N=114. Split my role. Table S2. HCPs views on Factors affecting physical activity support in clinic, split by role. [file 12887_2023_3940_MOESM1_ESM.docx]

| Table S1: HCPs views and attitudes towards supporting physical activity in clinics. N=114. Split my role | | | | | | | | | | | | | | | |
| --- | --- | --- | --- | --- | --- | --- | --- | --- | --- | --- | --- | --- | --- | --- | --- |
|  |  | Strongly agree, n (%) | |  | Agree, n (%) | |  | Neither agree nor disagree, n (%) | |  | Disagree, n (%) | |  | Strongly disagree, n (%) | |
| **Helping children and adolescents with type 1 diabetes to be physically active is part of my clinical role.** | Consultant | 18 (51%) | 74 (65%) | Consultant | 14 (40%) | 33  (29%) | Consultant | 3 (9%) | 5  (4%) | Consultant | 0 (0%) | 2  (2%) | Consultant | 0 (0% | 0  (0%) |
|  | Specialist nurse | 26 (67%) |  | Specialist nurse | 13(33%) |  | Specialist nurse | 0(0%) |  | Specialist nurse | 0 (0%) |  | Specialist nurse | 0 (0%) |  |
|  | Dietician | 28 (90%) |  | Dietician | 3 (10%) |  | Dietician | 0(0%) |  | Dietician | 0(0%) |  | Dietician | 0(0%) |  |
|  | Psychologist | 1 (13%) |  | Psychologist | 3 (38%) |  | Psychologist | 2 (25%) |  | Psychologist | 2 (25%) |  | Psychologist | 0(0%) |  |
|  | Associate specialist | 1 (100%) |  | Associate specialist | 0(0%) |  | Associate specialist | 0(0%) |  | Associate specialist | 0(0%) |  | Associate specialist | 0(0%) |  |
|  |  |  |  |  |  |  |  |  |  |  |  |  |  |  |  |
| Promoting physical activity in children and adolescents with type 1 diabetes is seen as important in the clinic that you work in. | Consultant | 18 (51%) | 65  (57%) | Consultant | 13(37%) | 37  (32%) | Consultant | 3 (9%) | 10  (9%) | Consultant | 1 (3%) | 2  (2%) | Consultant | 0(0%) | 0  (0%) |
|  | Specialist nurse | 25 (64%) |  | Specialist nurse | 10 (26%) |  | Specialist nurse | 3 (8%) |  | Specialist nurse | 1 (3%) |  | Specialist nurse | 0(0%) |  |
|  | Dietician | 20(65%) |  | Dietician | 10 (32%) |  | Dietician | 1 (3%) |  | Dietician | 0(0%) |  | Dietician | 0(0%) |  |
|  | Psychologist | 2 (25%) |  | Psychologist | 3 (38%) |  | Psychologist | 3 (38%) |  | Psychologist | 0(0%) |  | Psychologist | 0(0%) |  |
|  | Associate specialist | 0(0%) |  | Associate specialist | 1 (100%) |  | Associate specialist | 0(0%) |  | Associate specialist | 0(0%) |  | Associate specialist | 0(0%) |  |
|  |  |  |  |  |  |  |  |  |  |  |  |  |  |  |  |
| I don't advise children and adolescents with type 1 diabetes about physical activity unless specifically asked by the patient. | Consultant | 0(0%) | 1  (1%) | Consultant | 2 (6%) | 6  (5%) | Consultant | 2 (6%) | 5  (4%) | Consultant | 18 (51%) | 53 (46%) | Consultant | 13 (37%) | 49 (43%) |
|  | Specialist nurse | 1 (3%) |  | Specialist nurse | 2 (5%) |  | Specialist nurse | 0 (0%) |  | Specialist nurse | 16 (41%) |  | Specialist nurse | 20 (50%) |  |
|  | Dietician | 0(0%) |  | Dietician | 0 (0%) |  | Dietician | 2 (6%) |  | Dietician | 14 (45%) |  | Dietician | 15 (48%) |  |
|  | Psychologist | 0 (0%) |  | Psychologist | 2 (25%) |  | Psychologist | 1 (12.5%) |  | Psychologist | 4 (50%) |  | Psychologist | 1 (12.5%) |  |
|  | Associate specialist | 0 (0%) |  | Associate specialist | 0 (0%) |  | Associate specialist | 0 (0%) |  | Associate specialist | 1 (100%) |  | Associate specialist | 0 (0%) |  |
|  |  |  |  |  |  |  |  |  |  |  |  |  |  |  |  |
| I don't advise children and adolescents with type one diabetes about physical activity unless the patient reports difficulties with physical activity. | Consultant | 0 (0%) | 0 (0%) | Consultant | 1 (3%) | 6  (5%) | Consultant | 2 (6%) | 4  (4%) | Consultant | 18 (51%) | 57 (50%) | Consultant | 14 (40%) | 47 (41%) |
|  | Specialist nurse | 0(0%) |  | Specialist nurse | 3 (8%) |  | Specialist nurse | 0(0%) |  | Specialist nurse | 15 (38%) |  | Specialist nurse | 21 (54%) |  |
|  | Dietician | 0 (0%) |  | Dietician | 0 (0%) |  | Dietician | 1 (3%) |  | Dietician | 19 (61%) |  | Dietician | 11 (35%) |  |
|  | Psychologist | 0 (0%) |  | Psychologist | 2 (25%) |  | Psychologist | 1 (12.5%) |  | Psychologist | 4 (50%) |  | Psychologist | 1 (12.5%) |  |
|  | Associate specialist | 0 (0%) |  | Associate specialist | 0 (0%) |  | Associate specialist | 0 (0%) |  | Associate specialist | 1 (100%) |  | Associate specialist | 0 (0%) |  |
|  |  |  |  |  |  |  |  |  |  |  |  |  |  |  |  |
| **I have sufficient knowledge to advise children and adolescents with type 1 diabetes about physical activity.** | Consultant | 6 (17%) | 30 (26%) | Consultant | 23 (66%) | 62  (54%) | Consultant | 4 (11%) | 15  (13%) | Consultant | 2 (6%) | 5  (4%) | Consultant | 0(0%) | 2  (2%) |
|  | Specialist nurse | 9(23%) |  | Specialist nurse | 21 (54%) |  | Specialist nurse | 6 (15%) |  | Specialist nurse | 3 (8%) |  | Specialist nurse | 0(0%) |  |
|  | Dietician | 14 (45%) |  | Dietician | 15 (48%) |  | Dietician | 2 (6%) |  | Dietician | 0(0%) |  | Dietician | 0(0%) |  |
|  | Psychologist | 1 (12.5%) |  | Psychologist | 2 (25%) |  | Psychologist | 3(38%) |  | Psychologist | 0(0%) |  | Psychologist | 2 (25% |  |
|  | Associate specialist | 0(0%) |  | Associate specialist | 1 (100%) |  | Associate specialist | 0(0%) |  | Associate specialist | 0(0%) |  | Associate specialist | 0(0%) |  |
|  |  |  |  |  |  |  |  |  |  |  |  |  |  |  |  |
| **I try to encourage children and adolescents with type 1 diabetes to increase their physical activity levels.** | Consultant | 13 (37%) | 42 (37%) | Consultant | 20 (57%) | 58  (51%) | Consultant | 2 (6%) | 11  (10%) | Consultant | 0(0%) | 3  (3%) | Consultant | 0(0%) | 0  (0%) |
|  | Specialist nurse | 11 (28%) |  | Specialist nurse | 20 (51%) |  | Specialist nurse | 7 (18%) |  | Specialist nurse | 1(3%) |  | Specialist nurse | 0 (0%) |  |
|  | Dietician | 17 (55%) |  | Dietician | 14 (45%) |  | Dietician | 0(0%) |  | Dietician | 0(0%) |  | Dietician | 0(0%) |  |
|  | Psychologist | 1 (12.5%) |  | Psychologist | 3 (38%) |  | Psychologist | 2 (25%) |  | Psychologist | 2 (25%) |  | Psychologist | 0(0%) |  |
|  | Associate specialist | 0(0%) |  | Associate specialist | 1 (100%) |  | Associate specialist | 0(0%) |  | Associate specialist | 0(0%) |  | Associate specialist | 0(0%) |  |
|  |  |  |  |  |  |  |  |  |  |  |  |  |  |  |  |
| **When physical activity is discussed, advice is given to children and adolescents with type 1 diabetes on managing blood glucose.** | Consultant | 22 (63%) | 81 (71%) | Consultant | 13 (37%) | 30  (26%) | Consultant | 0(0%) | 3  (3%) | Consultant | 0(0%) | 0  (0%) | Consultant | 0(0%) | 0  (0%) |
|  | Specialist nurse | 34 (87%) |  | Specialist nurse | 5 (13%) |  | Specialist nurse | 0(0%) |  | Specialist nurse | 0 (0%) |  | Specialist nurse | 0 (0%) |  |
|  | Dietician | 20 (65%) |  | Dietician | 11 (35%) |  | Dietician | 0(0%) |  | Dietician | 0(0%) |  | Dietician | 0(0%) |  |
|  | Psychologist | 4 (50%) |  | Psychologist | 1 (12.5%) |  | Psychologist | 3 (38%) |  | Psychologist | 0(0%) |  | Psychologist | 0(0%) |  |
|  | Associate specialist | 1 (100%) |  | Associate specialist | 0(0%) |  | Associate specialist | 0(0%) |  | Associate specialist | 0(0%) |  | Associate specialist | 0(0%) |  |
|  |  |  |  |  |  |  |  |  |  |  |  |  |  |  |  |
| Blood glucose management during exercise is included as part of the ongoing education programme for children and adolescents with type 1 diabetes. | Consultant | 20(57%) | 76 (67%) | Consultant | 15 (43%) | 33  (29%) | Consultant | 0(0%) | 4  (4%) | Consultant | 0(0%) | 1  (1%) | Consultant | 0(0%) | 0  (0%) |
|  | Specialist nurse | 30(77%) |  | Specialist nurse | 5 (15%) |  | Specialist nurse | 2 (5%) |  | Specialist nurse | 1 (3%) |  | Specialist nurse | 0 (0%) |  |
|  | Dietician | 20 (65%) |  | Dietician | 10 (32%) |  | Dietician | 1 (3%) |  | Dietician | 0 (0%) |  | Dietician | 0 (0%) |  |
|  | Psychologist | 5 (63%) |  | Psychologist | 2 (25%) |  | Psychologist | 1 (12.5%) |  | Psychologist | 0 (0%) |  | Psychologist | 0 (0%) |  |
|  | Associate specialist | 1 (100%) |  | Associate specialist | 0 (0%) |  | Associate specialist | 0 (0%) |  | Associate specialist | 0 (0%) |  | Associate specialist | 0 (0%) |  |
|  |  |  |  |  |  |  |  |  |  |  |  |  |  |  |  |
| When physical activity is discussed, children and adolescents with type one diabetes are given a tailored blood glucose management plan. | Consultant | 3 (9%) | 25 (22%) | Consultant | 18 (51%) | 50  (44%) | Consultant | 11 (31%) | 29  (25%) | Consultant | 3 (9%) | 10  (9%) | Consultant | 0(0%) | 0  (0%) |
|  | Specialist nurse | 12 (31%) |  | Specialist nurse | 15(38%) |  | Specialist nurse | 8(21%) |  | Specialist nurse | 4(10%) |  | Specialist nurse | 0(0%) |  |
|  | Dietician | 9(29%) |  | Dietician | 12(39%) |  | Dietician | 8 (26%) |  | Dietician | 2 (6%) |  | Dietician | 0 (0%) |  |
|  | Psychologist | 1 (12.5%) |  | Psychologist | 4 (50%) |  | Psychologist | 2 (25%) |  | Psychologist | 1 (12.5%) |  | Psychologist | 0 (0%) |  |
|  | Associate specialist | 0 (0%) |  | Associate specialist | 1 (100%) |  | Associate specialist | 0 (0%) |  | Associate specialist | 0 (0%) |  | Associate specialist | 0 (0%) |  |

| Table S2: HCPs views on Factors affecting physical activity support in clinic, split by role. | | | | | | | | | | | | | | | |
| --- | --- | --- | --- | --- | --- | --- | --- | --- | --- | --- | --- | --- | --- | --- | --- |
|  |  | Strongly agree, n (%) | |  | Agree, n (%) | |  | Neither agree nor disagree, n (%) | |  | Disagree, n (%) | |  | Strongly disagree, n (%) | |
| I do not have enough time to discuss physical activity with children and adolescents with type one diabetes. | Consultant | 0 (0%) | 0 (0%) | Consultant | 9 (26%) | 16 (14%) | Consultant | 7 (20%) | 25 (22%) | Consultant | 16 (46%) | 54 (47%) | Consultant | 3 (9%) | 19 (17%) |
|  | Specialist nurse | 0 (0%) |  | Specialist nurse | 4 (10%) |  | Specialist nurse | 7 (18%) |  | Specialist nurse | 18 (46%) |  | Specialist nurse | 10 (26%) |  |
|  | Dietician | 0 (0%) |  | Dietician | 3 (10%) |  | Dietician | 8 (26%) |  | Dietician | 15 (48%) |  | Dietician | 5 (16%) |  |
|  | Psychologist | 0 (0%) |  | Psychologist | 0 (0%) |  | Psychologist | 3 (38%) |  | Psychologist | 4 (50%) |  | Psychologist | 1 (12.5%) |  |
|  | Associate specialist | 0 (0%) |  | Associate specialist | 0 (0%) |  | Associate specialist | 0 (0%) |  | Associate specialist | 1 (100%) |  | Associate specialist | 0 (0%) |  |
|  |  |  |  |  |  |  |  |  |  |  |  |  |  |  |  |
| Educational materials about physical activity are inappropriate for children and adolescents with type one diabetes. | Consultant | 0 (0%) | 4 (4%) | Consultant | 4 (11%) | 18 (16%) | Consultant | 14 (40%) | 33 (29%) | Consultant | 12 (34%) | 44 (39%) | Consultant | 5 (14%) | 15 (13%) |
|  | Specialist nurse | 2 (5%) |  | Specialist nurse | 7 (18%) |  | Specialist nurse | 9 (23%) |  | Specialist nurse | 16 (41%) |  | Specialist nurse | 5 (13%) |  |
|  | Dietician | 2 (6%) |  | Dietician | 7 (23%) |  | Dietician | 8 (26%) |  | Dietician | 11 (35%) |  | Dietician | 3 (10%) |  |
|  | Psychologist | 0 (0%) |  | Psychologist | 0 (0%) |  | Psychologist | 2 (25%) |  | Psychologist | 4 (50%) |  | Psychologist | 2 (25%) |  |
|  | Associate specialist | 0 (0%) |  | Associate specialist | 0 (0%) |  | Associate specialist | 0 (0%) |  | Associate specialist | 1 (100%) |  | Associate specialist | 0 (0%) |  |
|  |  |  |  |  |  |  |  |  |  |  |  |  |  |  |  |
| There is a lack of educational opportunities for health professionals regarding physical activity in children and adolescents with type one diabetes. | Consultant | 2 (6%) | 8 (7%) | Consultant | 13 (37%) | 42 (37%) | Consultant | 9 (26%) | 24 (21%) | Consultant | 11 (31%) | 37 (32%) | Consultant | 0 (0%) | 3 (3%) |
|  | Specialist nurse | 4 (10%) |  | Specialist nurse | 17 (44%) |  | Specialist nurse | 7 (18%) |  | Specialist nurse | 10 (26%) |  | Specialist nurse | 1 (3%) |  |
|  | Dietician | 1 (3%) |  | Dietician | 10 (32%) |  | Dietician | 4 (13%) |  | Dietician | 14 (45%) |  | Dietician | 2 (6%) |  |
|  | Psychologist | 1 (12.5%) |  | Psychologist | 2 (25%) |  | Psychologist | 4 (50%) |  | Psychologist | 1 (12.5%) |  | Psychologist | 0 (0%) |  |
|  | Associate specialist | 0 (0%) |  | Associate specialist | 0 (0%) |  | Associate specialist | 0 (0%) |  | Associate specialist | 1 (100%) |  | Associate specialist | 0 (0%) |  |
|  |  |  |  |  |  |  |  |  |  |  |  |  |  |  |  |
| Children and adolescents with type one diabetes are unlikely to be motivated to follow advice to be more active. | Consultant | 0 (0%) | 1 (1%) | Consultant | 4 (11%) | 17 (15%) | Consultant | 9 (26%) | 34 (30%) | Consultant | 20 (57%) | 47 (41%) | Consultant | 2 (6%) | 15 (13%) |
|  | Specialist nurse | 0 (0%) |  | Specialist nurse | 6 (15%) |  | Specialist nurse | 15 (38%) |  | Specialist nurse | 10 (26%) |  | Specialist nurse | 8 (21%) |  |
|  | Dietician | 0 (0%) |  | Dietician | 7 (23%) |  | Dietician | 8 (26%) |  | Dietician | 12 (39%) |  | Dietician | 4 (13%) |  |
|  | Psychologist | 1 (12.5%) |  | Psychologist | 0 (0%) |  | Psychologist | 2 (25%) |  | Psychologist | 4 (50%) |  | Psychologist | 1 (12.5%) |  |
|  | Associate specialist | 0 (0%) |  | Associate specialist | 0 (0%) |  | Associate specialist | 0 (0%) |  | Associate specialist | 1 (100%) |  | Associate specialist | 0 (0%) |  |
|  |  |  |  |  |  |  |  |  |  |  |  |  |  |  |  |
| **I feel confident giving children and adolescents with type one diabetes advice on managing blood glucose with physical activity.** | Consultant | 6 (7%) | 36 (32%) | Consultant | 22 (63%) | 57 (50%) | Consultant | 3 (9%) | 12 (11%) | Consultant | 4 (11%) | 6 (5%) | Consultant | 0 (0%) | 3 (3%) |
|  | Specialist nurse | 17 (44%) |  | Specialist nurse | 16 (41%) |  | Specialist nurse | 6 (15%) |  | Specialist nurse | 0 (0%) |  | Specialist nurse | 0 (0%) |  |
|  | Dietician | 12 (39%) |  | Dietician | 17 (55%) |  | Dietician | 2 (6%) |  | Dietician | 0 (0%) |  | Dietician | 0 (0%) |  |
|  | Psychologist | 1 (12.5%) |  | Psychologist | 1 (12.5%) |  | Psychologist | 1 (12.5%) |  | Psychologist | 2 (25%) |  | Psychologist | 3 (38%) |  |
|  | Associate specialist | 0 (0%) |  | Associate specialist | 1 (100%) |  | Associate specialist | 0 (0%) |  | Associate specialist | 0 (0%) |  | Associate specialist | 0 (0%) |  |
